# Bacterial and Archaeal Metagenome-Assembled Genome Sequences from Svalbard Permafrost

**DOI:** 10.1128/MRA.00516-19

**Published:** 2019-07-03

**Authors:** Yaxin Xue, Inge Jonassen, Lise Øvreås, Neslihan Taş

**Affiliations:** a Computational Biology Unit, Department of Informatics, University of Bergen, Bergen, Norway; b Department of Biological Sciences, University of Bergen, Bergen, Norway; c University Center in Svalbard, UNIS, Longyearbyen, Norway; d Earth and Environmental Sciences Area, Lawrence Berkeley National Laboratory, Berkeley, California, USA; e Biosciences Area, Lawrence Berkeley National Laboratory, Berkeley, California, USA; Portland State University

## Abstract

Permafrost contains one of the least known soil microbiomes, where microbial populations reside in an ice-locked environment. Here, 56 prokaryotic metagenome-assembled genome (MAG) sequences from 13 phyla are reported. These MAGs will provide information on metabolic pathways that could mediate biogeochemical cycles in Svalbard permafrost.

## ANNOUNCEMENT

Permafrost covers over 25% of the exposed land surface of the Northern Hemisphere and hosts a diversity of microbes proposed to be unique to cold habitats (1). These frozen soils contain a large reservoir of soil organic matter (SOM) that can have a significant impact on global climate upon thawing (2). The permafrost thaw may stimulate microbial activity and thus enable SOM decomposition. Previous studies have shown differences in microbial diversity between active layer (seasonally thawed and refrozen topsoil) and permafrost microbial communities (1–5). Although permafrost microbiomes are known to be highly diverse (1), they are largely underrepresented in global surveys. In this study, we investigated the microbial communities through a depth profile from Svalbard, and we report the binned metagenomic coassembly of five metagenome samples (6) and 56 metagenome-assembled genome (MAG) sequences.

Soil samples were obtained from an ice-wedge polygon site in the Adventdalen Valley in Svalbard, Norway (78.186N, 15.9248E). The site soil geochemistry was described previously (6). Five depth segments, namely, one active layer mineral horizon and four permafrost layers, were collected at the following depths: 0 to 14, 101 to 118, 118 to 126, 126 to 144, and 161 to 181 cm below the soil surface. Total community genomic DNA was extracted using a PowerSoil DNA isolation kit, and sequencing libraries were prepared using a TruSeq DNA library kit. An Illumina HiSeq 2500 instrument was used to acquire paired-end 150-bp metagenomic sequences, generating 20 Gb of raw reads per sample (7). The microbial community diversity and composition were reported elsewhere (6).

After adapter and low-quality reads were trimmed using MOCAT2 v2.0.0 (7), all cleaned reads were merged and then coassembled with MEGAHIT v1.1.3 (8), resulting in 566,254 contigs of ≥1 kb. We binned the contigs with MaxBin2 v2.2.5 (9) and MetaBAT2 v2.12.1 (10) and then dereplicated and aggregated them into MAGs using DAS Tool v1.1.0 (11), which resulted in 64 MAGs. We used CheckM v1.0.11 (12) to determine the completeness and contamination of these MAGs. We further examined the taxonomic distribution of contigs within each MAG based on Kaiju v1.6.2 (13) annotations and removed contaminating contigs. This process resulted in a total of 56 MAGs with contamination less than 10%. Default parameters were used with all software. We recovered 8 high-, 44 medium-, and 4 low-quality draft MAGs in accordance with minimum information about metagenome-assembled genome (MIMAG) standards (14). The MAGs were distributed across the following phyla: *Actinobacteria*, 11; *Proteobacteria*, 11; *Bacteroidetes*, 8; *Acidobacteria*, 7; *Chloroflexi*, 6; *Verrucomicrobia*, 4; *Saccharibacteria*, 2; *Gemmatimonadetes*, 2; candidate phylum *Dormibacteraeota* (AD3), 1; candidate phylum *Levybacteria*, 1; *Firmicutes*, 1; *Nitrospirae*, 1; and *Thaumarchaeota*, 1 (Table 1). Here, we report MAGs with 31.07 to 98.20% estimated completeness, and therefore the MAG sizes range from 731,988 to 5,534,727 bp. The MAGs will be used to investigate metabolic pathways that could impact SOM decomposition in permafrost soils. Results from the comparative genomic analyses of these MAGs will be published elsewhere.

**TABLE 1:** Detailed completeness and contamination results, genome size, GC content, MIMAG status, taxonomy, and ENA accession information of MAGs

MAG alias	Completeness (%)	Contamination (%)	Genome size (bp)	GC content (%)	MIMAG classification	Taxonomy^a^	ENA accession no.
Maxbin2.039_sub	98.2	9.2	3,147,504	55.5	Medium	*Acidobacteria* sp.	ERZ870056
Metabat.113	96.8	0.9	2,959,789	67.9	High	*Actinobacteria* sp.	ERZ870109
Metabat.158	96.6	2.4	4,406,707	63.9	High	*Alphaproteobacteria* sp.	ERZ870094
Metabat.151	96.4	5.1	4,482,786	36.9	Medium	*Bacteroidetes* sp.	ERZ870080
Metabat.89	96.3	2.2	2,753,811	53.6	High	*Verrucomicrobia* sp.	ERZ870097
Metabat.179	95.3	0.9	2,724,314	69.2	High	*Chloroflexi* sp.	ERZ870110
Metabat.143	94.4	2.0	2,442,640	66.0	High	*Chloroflexi* sp.	ERZ870099
Metabat.177_sub	94.3	6.7	4,572,140	59.2	Medium	*Proteobacteria*	ERZ870074
Metabat.40	93.6	3.4	3,692,750	65.4	High	*Betaproteobacteria*	ERZ870086
Metabat.123_sub	93.2	9.6	4,243,256	68.2	Medium	*Actinobacteria* sp.	ERZ870064
Metabat.14	92.6	3.9	2,553,466	66.1	High	*Chloroflexi* sp.	ERZ870083
Metabat.133	91.6	1.9	2,305,255	67.3	High	Candidate *Dormibacteraeota* sp.	ERZ870101
Metabat.147	91.3	7.9	4,040,741	55.9	Medium	*Verrucomicrobia* sp.	ERZ870070
Metabat.67	89.9	5.5	1,906,190	68.3	Medium	*Actinobacteria* sp.	ERZ870077
Maxbin2.041	89.7	2.2	3,901,541	59.3	Medium	*Acidobacteria* sp.	ERZ870096
Metabat.164_sub	89.4	4.7	2,849,413	64.2	Medium	*Chloroflexi* sp.	ERZ870081
Maxbin2.071_sub	86.5	8.3	3,144,416	70.1	Medium	*Actinobacteria* sp.	ERZ870067
Metabat.51	85.9	8.2	2,827,458	60.8	Medium	*Gemmatimonadetes* sp.	ERZ870069
Maxbin2.021_sub	85.7	6.8	2,132,093	70.0	Medium	*Chloroflexi* sp.	ERZ870073
Metabat.154	84.8	2.5	2,330,430	69.6	Medium	*Actinobacteria* sp.	ERZ870091
Metabat.156	84.7	1.5	2,372,385	35.6	Medium	*Bacteroidetes* sp.	ERZ870107
Maxbin2.102_sub	84.6	1.8	2,720,713	64.2	Medium	*Acidobacteriaceae* sp.	ERZ870102
Metabat.138	84.4	2.0	2,813,002	55.1	Medium	*Verrucomicrobia* sp.	ERZ870098
Metabat.172_sub	83.2	2.4	2,237,822	65.1	Medium	*Rhizobiales* sp.	ERZ870093
Maxbin2.128	82.1	9.8	2,270,224	51.7	Medium	*Alphaproteobacteria* sp.	ERZ870062
Maxbin2.086_sub	81.9	9.7	3,605,629	57.9	Medium	*Acidobacteria* sp.	ERZ870063
Metabat.159_sub	81.7	8.7	2,099,345	55.9	Medium	*Verrucomicrobia* sp.	ERZ870066
Metabat.121	80.2	3.5	2,452,147	35.8	Medium	*Bacteroidetes* sp.	ERZ870085
Metabat.122	77.3	8.3	2,004,053	67.9	Medium	*Actinobacteria* sp.	ERZ870068
Metabat.163_sub	73.8	2.0	2,166,091	71.1	Medium	*Solirubrobacterales* sp.	ERZ870100
Metabat.72	72.9	3.2	3,967,186	40.8	Medium	*Bacteroidetes* sp.	ERZ870087
Metabat.167	72.5	2.3	2,102,822	70.7	Medium	*Actinobacteria* sp.	ERZ870095
Metabat.115	72.1	5.1	1,795,856	70.2	Medium	*Actinobacteria* sp.	ERZ870079
Metabat.174	71.6	2.5	2,317,750	35.4	Medium	*Bacteroidetes* sp.	ERZ870092
Metabat.53	71.3	8.2	5,534,727	37.1	Medium	*Bacteroidetes* sp.	ERZ879091
Metabat.100	69.8	0.9	2,344,086	68.8	Medium	*Solirubrobacterales* sp.	ERZ870111
Metabat.26	67.9	0.8	2,094,082	68.3	Medium	*Actinobacteria* sp.	ERZ870112
Metabat.119	67.2	0.0	731,988	47.4	Medium	*Saccharibacteria* sp.	ERZ870115
Metabat.140	67.1	6.0	1,381,010	69.0	Medium	*Chloroflexi* sp.	ERZ870075
Metabat.16	66.2	1.5	844,132	41.3	Medium	*Thaumarchaeota* sp.	ERZ870108
Maxbin2.015	65.5	4.0	2,138,105	49.3	Medium	*Proteobacteria* sp.	ERZ870082
Maxbin2.090	64.2	5.9	2,561,445	65.2	Medium	*Gemmatimonadetes* sp.	ERZ870076
Metabat.48	63.6	1.7	741,844	38.9	Medium	Candidate *Levybacteria* sp.	ERZ870104
Metabat.28	63.5	2.6	2,845,538	67.0	Medium	*Burkholderiales* sp.	ERZ870090
Metabat.166	63.3	0.2	739,124	45.6	Medium	*Saccharibacteria* sp.	ERZ870114
Maxbin2.012	63.2	6.9	2,750,113	55.1	Medium	*Proteobacteria* sp.	ERZ870072
Metabat.12	63.0	1.6	2,221,067	39.3	Medium	*Bacteroidetes* sp.	ERZ870106
Metabat.155_sub	58.6	2.9	1,479,786	56.8	Medium	*Nitrosomonadales* sp.	ERZ870089
Metabat.94	58.2	3.1	3,546,342	59.7	Medium	*Acidobacteria* sp.	ERZ870088
Maxbin2.095_sub	53.4	8.8	2,850,869	56.6	Medium	*Nitrospirae* sp.	ERZ870065
Metabat.1	51.9	0.6	1,114,730	51.0	Medium	*Nitrosospira* sp.	ERZ870113
Metabat.170	51.4	3.6	3,578,256	59.6	Medium	*Acidobacteria* sp.	ERZ870084
Metabat.175	48.3	1.6	1,833,825	41.8	Low	*Bacteroidetes* sp.	ERZ870105
Maxbin2.011	42.4	5.2	2,493,859	62.5	Low	*Rhizobiales* sp.	ERZ870078
Maxbin2.064_sub	40.9	7.4	1,652,927	43.4	Low	*Firmicutes* sp.	ERZ870071
Maxbin2.096_sub	31.1	1.8	1,233,990	54.5	Low	*Acidobacteria* sp.	ERZ870103

a Uncultured isolates were used.

### Data availability.

The shotgun sequence data were deposited in the European Nucleotide Archive (ENA) database under the study number PRJEB30872 with the accession numbers ERR3078909 to ERR3078913. The MAGs are publicly available in the ENA under the analysis accession numbers ERZ870056, ERZ870062 to ERZ870115, and ERZ879091.
